# Imatinib-Induced Myopathy in a Patient With Gastrointestinal Stromal Tumor: A Case Report

**DOI:** 10.7759/cureus.90251

**Published:** 2025-08-16

**Authors:** Max A Vogel, Liam P White, Ross M Michels

**Affiliations:** 1 Oncology, University of South Carolina School of Medicine Greenville, Greenville, USA

**Keywords:** acute inflammatory demyelinating polyneuropathy, gastrointestinal stromal tumor, imatinib, immunotherapy, myositis

## Abstract

Gastrointestinal stromal tumors (GISTs) are a neoplasm derived from interstitial cells of Cajal in the gastrointestinal tract. These tumors arise secondary to mutations in the KIT tyrosine kinase gene. First-line treatment for GISTs is imatinib, a selective tyrosine kinase inhibitor that blocks the function of the aberrant protein that drives the tumor. Imatinib is generally well-tolerated, with the most common adverse effects including nausea/vomiting, diarrhea, and edema.

We present a case of a 68-year-old man who developed acute inflammatory demyelinating polyneuropathy (AIDP) with superimposed myositis following treatment of BRAF-wild type GIST with imatinib. Although myopathic adverse effects have been documented with other tyrosine kinase inhibitors, myopathy as an adverse effect of imatinib is rarely documented in the literature. This case highlights a rare but significant neuromuscular complication potentially linked to imatinib and emphasizes the importance of prompt recognition, thorough diagnostic workup, and interdisciplinary management.

## Introduction

Gastrointestinal stromal tumors (GISTs) are an uncommon type of neoplasm, arising from interstitial cells of Cajal in the gastrointestinal tract [1]. The majority of GISTs, around 80%, arise from mutations in the KIT gene, with around 5% arising from activating mutations in the platelet-derived growth factor receptor alpha (PDGFRA) gene. Each mutation causes constitutive activation of receptor tyrosine kinase, resulting in ligand-independent transduction of signals, leading to cellular proliferation [2,3]. First-line medical management of GISTs utilizes imatinib, a selective tyrosine kinase inhibitor that blocks the function of KIT- and PDGFRA-associated tyrosine kinases. The drug is dosed at 400 mg daily or 400 mg twice daily, depending on disease response and patient tolerance of the medication. Imatinib is effective and generally well-tolerated, although most patients experience at least one grade 1 or 2 mild to moderate adverse event [1].

The most common adverse events associated with imatinib administration in GIST patients are gastrointestinal complaints and edema. Studies have shown complaints of nausea/vomiting or diarrhea in over 50% of patients, and edema in over 70% of patients. Other well-documented adverse reactions include cutaneous manifestations such as rash/dermatitis, fatigue, muscle cramping, myalgia/arthralgia, and headache [1]. Severe events, including decompensation of heart failure, gastrointestinal bleeding, bone marrow suppression, and hepatotoxicity, are infrequent but well-documented adverse effects [4]. Myositis has been documented as an adverse event associated with other tyrosine kinase inhibitors, but myopathic events related to imatinib are poorly documented. Previous case reports suggest myopathic pathologies such as dermatomyositis and inclusion body myositis as possible complications of therapy [4,5]. While such cases are documented in the literature, severe myopathic adverse effects of imatinib are exceedingly rare. In this case, we present a patient who developed acute inflammatory demyelinating polyneuropathy (AIDP) with superimposed myositis following treatment of GIST with imatinib.

## Case presentation

A 68-year-old male underwent a routine colonoscopy in 2023, which revealed a 6 cm extraluminal rectal sidewall mass. Following endoscopic ultrasound-guided biopsy, the patient was diagnosed with a BRAF wild-type GIST tumor. Staging imaging following diagnosis was negative for metastatic disease, and the patient was started on a course of neoadjuvant imatinib six months later. His original treatment course was dosed at 400 mg per day. Initial therapy was tolerated poorly due to anasarca. Therapy was held at the beginning of 2024 in anticipation of surgical resection of his tumor. Robotic abdominoperineal resection with end colostomy was completed four months later.

Following surgery, the patient was restarted on imatinib at a 50% dose reduction from the original treatment regimen. Two months postoperatively, and within two weeks of restarting imatinib, the patient reported an acute onset of lower extremity weakness. Weakness was most pronounced in the proximal muscle groups of the lower extremities. At the visit, the patient was unable to ambulate without assistance due to weakness. Neurologic examination demonstrated 1/5 strength in bilateral iliopsoas muscles, 4/5 strength in bilateral hamstring muscles, 4/5 strength in bilateral foot dorsiflexion, and 4/5 strength in the bilateral deltoids. Sensation to light touch and pinprick was reduced bilaterally in the lower extremities but intact in the upper extremities. The remainder of the neurological exam was within normal limits.

The creatine kinase level at this time was above 4,000. Imatinib was held at this time. The patient was subsequently admitted to the hospital and evaluated by neurology. At presentation to the hospital, he was diffusely areflexic, with extremity weakness most pronounced in the lower extremities. Laboratory work showed elevated inflammatory markers, hyperkalemia, and elevated creatine kinase (Table 1). Creatine and potassium are commonly elevated as damaged muscle fibers break down and release intracellular components. These laboratory evaluations led neurology to initiate a workup for a myopathic process.

**Table 1:** Pertinent Laboratory Results *Clinically significant and suggestive of myopathy Note: The patient had baseline creatinine elevation due to pre-existing renal disease. Hemoglobin was consistent with baseline, as seen on routine labs with the primary oncologist.

Lab	Value	Reference Range
Leukocytes (K/uL)	5.7	3.5-10.8
Hemoglobin (M/uL)	9.3	12.7-17.2
Mean corpuscular volume (fL)	93.7	79.0-100.0
Mean corpuscular hemoglobin (pg)	29.4	25.7-33.2
Potassium (mmol/L)*	5.6	3.5-5.1
Chloride (mmol/L)	107	98-107
Sodium (mmol/L)	140	136-145
C-reactive protein (mg/L)*	23.1	<5
Creatinine (mg/dL)	3.11	0.67-1.17
Aspartate aminotransferase (IU/L)	20	10-50
Alanine aminotransferase (IU/L)	6	10-50
Creatinine kinase (U/L)	4703	41-331

Nerve conduction studies and electromyography were subsequently obtained and demonstrated myopathic units consistent with irritable myopathy of the proximal muscle groups of the lower extremities. In addition, these studies noted generalized, symmetric, sensory and motor axonal, and demyelinating polyneuropathy meeting diagnostic criteria for AIDP. Based on the workup, the most likely etiology was thought to be AIDP with superimposed myositis secondary to imatinib administration.

The patient was admitted to the hospital at that time. Creatine kinase levels were repeated on admission, demonstrating a marked decrease following cessation of imatinib. An MRI of the lower extremities obtained after hospitalization demonstrated symmetric muscular edema consistent with myositis. A myomarker three-panel was also obtained to screen for antibodies indicative of dermatomyositis, polymyositis, and antisynthetase syndrome (Table 2). All antibodies screened with this test returned negative.

**Table 2:** Myomarker Three Panel Note: Antibodies reported with a numerical value of <20 units are considered negative.

Lab	Value
Anti-Jo-1 antibody (units)	<20
Anti-PL antibody	Negative
Anti-PL-12 antibody	Negative
Anti-EJ antibody	Negative
Anti-OJ antibody	Negative
Anti-SRP antibody	Negative
Anti-TIF-1gamma antibody	Negative
Anti-MDA-5 antibody (units)	<20
Anti-NXP-2 antibody (units)	<20
Anti-PM/Scl-100 antibody (units)	<20
Anti-Ku antibody	Negative
Anti-SS-A 52kD antibody (units)	<20
Anti-U1 RNP antibody (units)	<20
Anti-U2 RNP antibody	Negative
Anti-U3 RNP antibody	Negative

**Figure 1:**
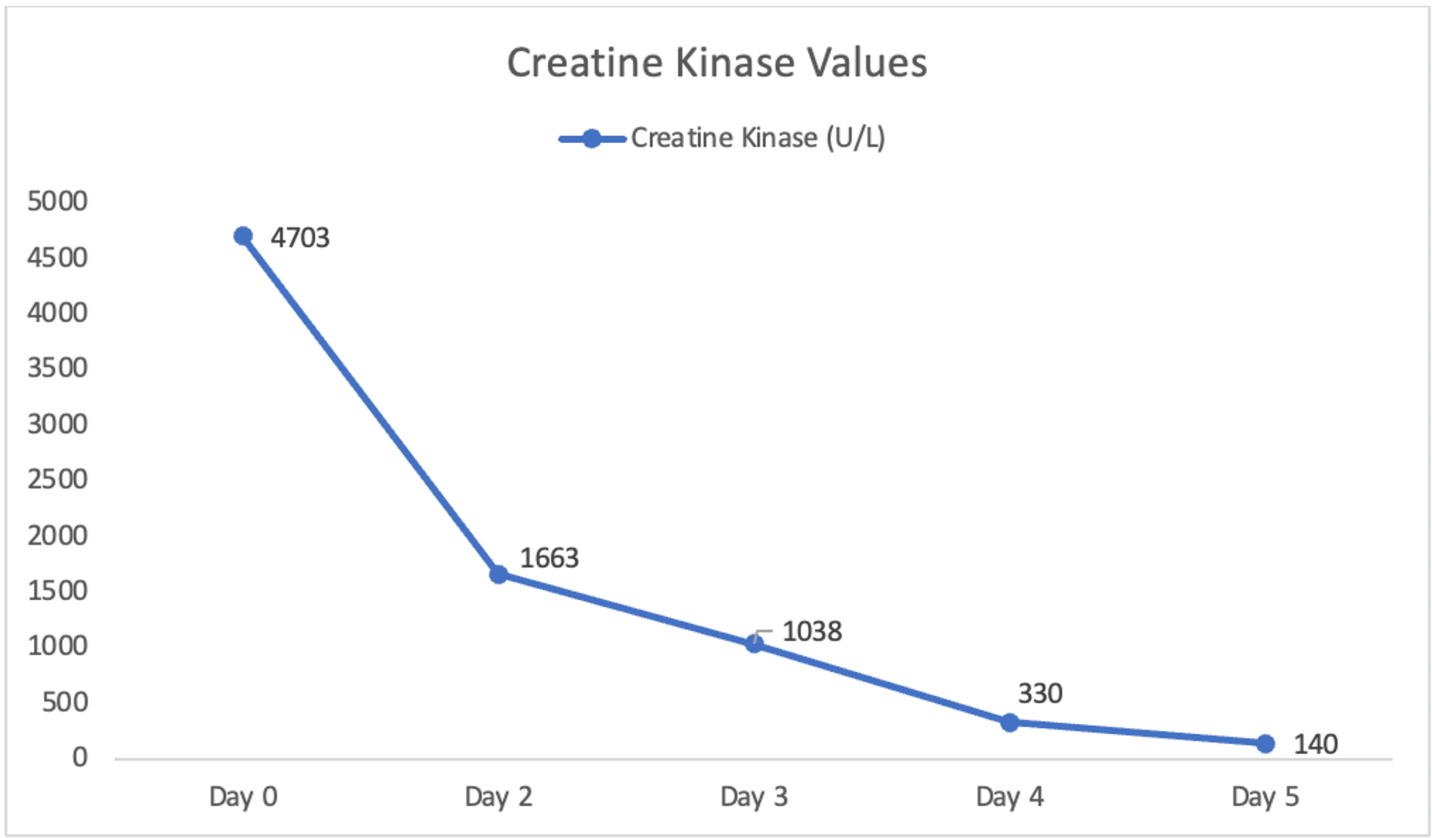
Creatine Kinase Values Note: Imatinib was held, and corticosteroids were initiated on Day 0. Plasmapheresis was initiated following the discontinuation of creatine kinase monitoring.

During hospitalization, the patient underwent two sessions of plasmapheresis and a course of Solu-Medrol. While intravenous immunoglobulin (IVIG) therapy was considered for treatment of suspected AIDP, plasmapheresis was selected due to concern for volume overload in the setting of the patient’s underlying renal disease. With treatment, the patient’s symptoms improved, and creatine kinase normalized. Creatine kinase values during hospitalization can be found in Figure 1.

The patient underwent three additional sessions of plasmapheresis in the outpatient setting, for a total of five sessions. Following completion of treatment, the patient's strength returned to near baseline. No further relapses occurred following cessation of plasmapheresis.

Following resolution of neurologic symptoms, the patient was started on sunitinib, which was complicated by nephrotic syndrome and acute kidney injury. He remains on surveillance with imaging every three months and has no recurrent disease to date. 

## Discussion

Although adverse effects related to the musculoskeletal system have been well-documented with imatinib, they are generally mild (grade 1 or 2) and benign. Rare reports of myopathic adverse effects have been documented in the literature. Silva et al. (2024) documented a case in which a patient being treated with imatinib for chronic myeloid leukemia presented with lower limb myalgia, inflammatory polyarthralgia, and weakness of the thighs. Initial testing was suggestive of an inflammatory myopathy, with elevated creatine kinase, acute phase reactants, and mild anemia noted on laboratory testing. The patient was noted to be positive for antinuclear antibodies and anti-Mi2a antibodies, suggestive of dermatomyositis. MRI and muscle biopsy results confirmed dermatomyositis, although the patient lacked typical cutaneous manifestations associated with this diagnosis [5].

In contrast to the aforementioned case, the patient presented in this report was not screened for anti-Mi2a antibodies. Screening for other dermatomyositis-associated antibodies was negative on myomarker-3 testing, suggesting an alternate etiology. Other myopathic conditions have been anecdotally reported in the literature, including a case of inclusion-body myositis in a patient treated for GIST with imatinib, although this patient was treated for a significantly longer duration than the patient presented in this case [4].

Certain tyrosine kinase inhibitors, such as osimertinib, have also been associated with elevated creatine kinase and myositis. Several case reports exist in the literature describing patients treated with osimertinib, featuring either asymptomatic elevations in creatine kinase or elevated creatine kinase found after patients reported muscle cramping [6,7].

A case described by Queirós Coelho et al. (2024) details a rapidly progressive generalized myopathy with limb weakness, ataxia, ptosis, dysarthria, dysphonia, and dysphagia 30 days after starting axitinib for clear cell renal cell carcinoma. This patient had similarly elevated creatine kinase as the patient described in this case report at presentation (4226 vs. 4703) and also had electromyography demonstrating inflammatory myopathy. Unlike the patient described in this case study, the patient presented by Queirós Coelho et al. (2024) was on pembrolizumab, an immune checkpoint inhibitor that may have caused or exacerbated myopathy [8].

Although these reports have similarities to the patient described in this case study, osimertinib and axitinib primarily act on epidermal growth factor receptor tyrosine kinases, thus working through a different mechanism of action than imatinib. Case reports exist of neuropathy-induced paralysis in patients treated with other PDGFRA-associated tyrosine kinases, such as sunitinib. These cases differ significantly from the presentation seen in this case study, including a case of Guillain-Barré syndrome following treatment of GIST with sunitinib [9].

In the case described in this report, there were several limitations to the patient’s diagnostic workup. Nerve conduction studies and electromyography were consistent with AIDP with superimposed myositis, and resolution of symptoms after initiation of plasmapheresis and immune suppression with corticosteroids suggests an immune-mediated phenomenon. Spinal MRI and cerebrospinal fluid analysis were not conducted to confirm the patient’s suspected AIDP. In addition, no muscle biopsy was obtained for characterization of suspected myositis, and an antibody screen for dermatomyositis, polymyositis, and antisynthetase syndrome, obtained during hospitalization, was negative.

Although this case shares similarities with other cases of tyrosine kinase-induced myopathic or demyelinating disorders, the exact characterization of the causative etiology was limited by the diagnostic workup. Imatinib has known skeletal muscle toxicity, often manifested as myalgia and muscle cramps. Previous studies have demonstrated that imatinib has the potential to disrupt mitochondrial function through inhibition of oxidative phosphorylation. One such study demonstrated that imatinib inhibited the activity of complex I in the mitochondrial respiratory chain in human rhabdomyosarcoma cells, leading to accumulation of reactive oxygen species and subsequent apoptosis and atrophy [10].

A similar mechanism could serve as a potential explanation for the myopathic adverse effects observed in this case report, including the muscle weakness and laboratory findings consistent with rhabdomyolysis. This mechanism would not explain the demyelination observed in the patient’s electromyogram and nerve conduction studies.

A paraneoplastic syndrome from the tumor itself was also considered, given this patient’s symptoms. GIST has been selectively marked by CD117, also found in the interstitial cells of Cajal, which are located throughout the muscular layer of the gastrointestinal tract and are neural-related [11]. There have been isolated reports in the literature regarding myopathic and neurologic paraneoplastic syndromes associated with GIST. A case of acute anti-Mi-2 antibody dermatomyositis was observed, in which workup yielded a GIST, with resolution of myopathic symptoms after multiple steroid treatments and tumor resection [11]. Vital et al. (2009) identified a case of acute sarcoid neuropathy four months after resection of GIST, with no other manifestations of sarcoidosis or indication of inflammatory myopathy [12].

Paraneoplastic etiology is not suspected in our patient’s case, as symptoms resolved immediately after cessation of imatinib and did not recur following immunosuppressive therapy and plasmapheresis. The features of acute creatine kinase elevation and improvement with plasmapheresis are not congruent with neurosarcoidosis presentation status post-resection. The Naranjo Adverse Drug Reaction Scale is a widely accepted causality assessment tool used in the evaluation of suspected adverse drug reactions, classifying reactions as definite, probable, possible, or doubtful [13]. This scale was used to assess the likelihood that imatinib was the causative factor in the symptoms observed in this case report. Based on this causality assessment tool, imatinib was the probable cause of this patient’s observed symptoms. Despite diagnostic limitations associated with this case, the observed adverse effects were likely linked to imatinib.

## Conclusions

Although myositis has been documented as a side effect of other tyrosine kinase inhibitors, imatinib has rarely been cited as a causative agent. Clinicians prescribing imatinib should consider monitoring creatine kinase and complete neurologic evaluation in patients presenting with new-onset weakness. These initial evaluations would allow classification of the seemingly diverse symptom presentation into a specific myositis or demyelinating etiology. This information would allow further research to establish a potential correlation with imatinib and other tyrosine kinase inhibitors. Appropriate workup for AIDP and myositis, including spinal MRI, cerebrospinal fluid studies, and muscle biopsy, was notably absent from this case. Due to concern for volume overload in the setting of underlying renal dysfunction, treatment with plasmapheresis was elected without pursuing further biopsy, imaging, or serologic studies. This makes it difficult to ascertain the specific etiology of this patient’s myopathic pain, further highlighting the need for a complete diagnostic workup, including muscle biopsy, cerebrospinal fluid analysis, and imaging, if a similar side effect profile is encountered in the future.
